# Involving frail older patients in identifying outcome measures for transitional care—a feasibility study

**DOI:** 10.1186/s40900-021-00288-9

**Published:** 2021-06-03

**Authors:** Troels Kjærskov Hansen, Annesofie Lunde Jensen, Else Marie Damsgaard, Tone Maria Mørck Rubak, Mikkel Erik Juul Jensen, Merete Gregersen

**Affiliations:** 1 Department of Geriatrics, Aarhus University Hospital, Aarhus, Denmark; 2 ResCenPi – Research Centre for Patient Involvement, Aarhus University & the Central Denmark Region, Aarhus, Denmark

**Keywords:** Patient and public involvement, Patient-reported outcome measures, Core outcome sets, Elderly, Relatives, Frailty, Transitional care

## Abstract

**Background:**

During care transitions, the older (75+) patient’s agenda can easily be missed. To counteract this, involving patients in shared clinical decision making has proven to be of great value. Likewise, involving patients and other stakeholders as researchers is gaining ground. Patient and public involvement (PPI) in research entails many benefits, for example, by bringing further insight from those with lived experiences of being ill. There are various challenges associated with involving some older patients, for example frailty, cognitive impairment and other chronic illnesses. To the best of our knowledge, there are only a few examples of initiatives involving older patients beyond research participation. The feasibility of involving frail older patients during an ongoing care transition from hospital to primary health care remains unknown. To investigate the feasibility of including older frail patients, their relatives and health care professionals (HCPs) as co-researchers, we established a study with increasingly demanding levels of patient involvement to identify relevant outcome measures for future transitional care research.

**Methods:**

The study was a pragmatic, qualitative feasibility study. The involved individuals were frail older patients, their relatives and HCPs. Patients and their relatives were interviewed, while the interviewer made reflective notes. A thematic analysis was made. Relatives and HCPs discussed the themes to identify relevant outcome measures and potentially co-create new patient-reported outcome measures (PROMs) for use in future transitional care studies. The feasibility was evaluated according to six involvement steps. The level of involvement was evaluated using the five-levelled Health Canada Public Involvement Continuum (HCPIC).

**Results:**

In total, eight patients, five relatives and three HCPs were involved in the study. Patients were involved in discussing care transitions (HCPIC level 3), while some relatives were engaged (HCPIC level 4) in forming PROMs. The partnership level of involvement (HCPIC level 5) was not reached. The thematic analysis and the subsequent theme discussion successfully formed PROMs. The key PROMs were related to care, transparency and the relatives’ roles in the transitional care process.

**Conclusions:**

When applying a pragmatic involvement approach, frail older patients can be successfully involved in identifying relevant transitional care outcome measures; however, involving these patients as fellow researchers seems infeasible. To maintain involvement, supportive relatives are essential. Useful experiences for future research involvement of this vulnerable group were reported, arguing that patient participation has the potential to become inherent in future geriatric research.

**Supplementary Information:**

The online version contains supplementary material available at 10.1186/s40900-021-00288-9.

## Plain English summary

The purpose of the study was to involve patients in identifying relevant outcome measures for future transitional care research. Involving patients in research is not new. What makes this project special is that it seeks to involve old, frail patients aged 75 plus.

The project used open-form interviews that were not constrained in time and were not audio recorded; this was done to obtain confidence from the patients and their relatives. Each patient was interviewed twice: shortly before the patients left the hospital and shortly after discharge. The purpose was to discuss the patient’s experiences during the discharge period. The first interview took place in the hospital, whereas the interviewer visited the patients in their residence for the second interview. An expert panel was then formed involving the patients’ relatives and the professional health care workers. The expert panel discussed themes based on the data expressed by the patients during the interviews. In addition, an attempt was made to establish long-term cooperation between the patients, their relatives and the researchers. The health condition and vulnerable state of the patients made it difficult to continue their involvement throughout the research process. In fact, only the relatives and professionals were able to take part in the expert panel. Despite these challenges, the outcome of the project was positive. In conclusion, it makes sense to involve frail patients in transitional care research despite the challenges these patients face in their old age. In future research, frail older patients, relatives and other stakeholders can be involved.

**Supplementary Information:**

The online version contains supplementary material available at 10.1186/s40900-021-00288-9.

## Background

Involving older patients and their family members in shared decision making and clinical care has been described in a variety of setups [1–5], and numerous reviews describe how older patients can be involved in research [6–8]. However, it remains unsettled how to best involve older, frail patients in geriatric research processes [9]. Care transitions of geriatric patients normally involve a number of different players with possibly different agendas. The agenda of the patient can easily be missed [3, 5, 10, 11]. Combining the need for promoting the older patients’ point of view on care transitions and for studying the potential for involvement of frail older patients in research, we designed an interview- and panel-based collaborative research study seeking to involve frail older patients, their relatives and geriatric health care professionals (HCPs).

Changing the role of the patient in research towards active involvement may come with numerous beneficial consequences: improved research quality, facilitation of the translation of study results and democratisation of the research process [12]. Involving marginalised groups [12] as well as older people [7] may even have an empowering effect on the involved people [13, 14]. Generally, involving patients in research is considered feasible [12]. Indeed, older people are able and willing to be involved in research [7, 10, 14, 15], and the potential barriers to involving older people are similar to the barriers identified in studies involving younger people [7]. Even involvement of older care-home residents is possible when taking the barriers and facilitators of involvement into consideration [6, 8].

Various PPI-supportive advisory groups define (patient and public) involvement in overlapping ways [16–18]. The world of health and social care research and the world of policy and service development have developed their own tools and terminologies. The British National Institute for Health Research (NIHR) advisory group INVOLVE supports public involvement in health and care research. INVOLVE define PPI as ‘research being carried out “with” or “by” members of the public rather than “to”, “about” or “for” them’ [18]. PPI can be applied in multiple ways and occasions during the research process. There are different approaches to involvement ranging from consultation through to co-production [18]. The involved should preferably be people with relevant lived experience of the health condition being researched [18, 19]. Traditionally involvement is perceived as being a distinct activity from participation, with the latter referring to patients having data collected from them [18, 19]. The Canadian Institutes of Health Research’s (CIHR) “Health Canada Policy Toolkit for Public Involvement in Decision Making” [16, 20] supports involvement of citizens in government decision making on health issues. The CIHR describe involvement in a five-leveled continuum ranging from one-way communications to collaborative decision-making and partnering as described in the Health Canada Public Involvement Continuum (HCPIC) [16]. The five HCPIC-levels are as follows: 1) inform/educate; 2) gather information; 3) discuss; 4) engage; and 5) partner. Seen through this lens, users of health services and study participants can be involved as fellow decision makers.

Despite the advantages of PPI, involving patients in research still has not yet fulfilled its potential in the geriatric research field. Geriatric patients are not only older but often frail and, in some cases, nearing death. Defining frailty as ‘a state of increased vulnerability to poor resolution of homoeostasis after a stressor event, which increases the risk of adverse outcomes, including falls, delirium, and disability [21], frail patients may likely face particular challenges that need to be overcome to accommodate their involvement in research, and their relatives may play a significant role in this [3]. The Multidimensional Prognostic Index (MPI) [22–24] provides a standardised frailty measure expressed in an aggregated score (range: 0–1) including information on eight domains: functional status (Functional Recovery Score Activities of Daily Living, Functional Recovery Score Instrumental Activities of Daily Living), cognitive status (Short Portable Mental Status Questionnaire), nutritional status (Mini Nutritional Assessment Short Form), mobility and risk of pressure sore (Exton-Smith Score), multimorbidity (Cumulative Illness Rating Scale Geriatrics), polypharmacy and cohabitation status. From our combined researchers’ and clinicians’ point of view, it seemed doubtful whether frail older patients would be able to be involved in all stages of the research: research planning, managing, designing and execution [18, 19]. Nonetheless, many frail patients are fully capable of forming clear opinions on important matters regarding their own treatment and care. Still, the feasibility of involving frail, older patients exposed to stressful events such as care transitions remains unknown. To investigate how and to what extent involving frail older patients in research is possible, we aimed to establish a research project allowing patients and other stakeholders to be gradually involved in the process. For this purpose, the CIHR definition of involvement and the HCPIC was appropriate, looking upon involvement as a continuum.

In frailty and transitional care research, outcome measures such as a lack of mortality and readmissions are commonly used by researchers as markers of high-quality care although patients and caregivers may prefer other outcome measures, for example, care continuity and medication management [25–28]. The International Consortium for Health Outcomes Measurement (ICHOM) [29] presents patient-centred outcome standard sets for a wide range of conditions and populations, including older patients [30] and people living with dementia [31]. The most important outcomes may be assessed by using patient-reported outcome measures (PROMs), such as the Short Form 36 (SF-36) that measures one’s quality of life [32], UCLA 3-item Loneliness Scale measuring loneliness [33, 34] and Zarit Burden Interview 4-item screening questionnaire that measures the caregiver’s burden [35]. Other workgroups have proposed outcomes yielding desired caregiver and patient outcomes [25, 27]. However, to the best of our knowledge, no specific set of PROMs or core outcome sets (COS) [28] for frail patients exists. Similarly, knowledge regarding the outcomes of the greatest importance to patients specifically during care transition is limited [5, 10, 27].

The objective of the current study was to investigate the extent to which patients and relatives were willing and able to be involved as fellow transitional care researchers while seeking relevant transitional care outcome measures and investigating frail older patients’ views on care transitions.

## Methods

### Design

The current study was a pragmatic, qualitative feasibility study [36] comprising interviews, thematic analysis and panel-based discussions; the study was designed to involve frail older patients and their relatives to the highest possible HCPIC-level of involvement, ideally forming a transitional care PPI panel for future research projects. The research was conducted pre-Covid.

### People involved

The individuals invited to be involved were a group of patients, some of their relatives and a small predefined group of three HCPs. Eligible patients were frail, hospitalised because of acute illness and aged 75 years or older. Given the study’s explorative nature, we considered nine or more patients and relatives and a minimum of two HCPs to be enough to attain sufficient information power [37]. The HCPs (MEJJ, EMD, MG) were all involved in designing the study, and one of them (MEJJ) was also involved in finding and inviting eligible patients. All patients were admitted to the geriatric ward at Aarhus University Hospital between February 5 and March 15, 2019. The ward consists of 32 single-bed rooms and includes geriatric patients admitted with stroke, orthopaedic and medical conditions. Patients undergoing palliative care, stroke and orthopaedic treatment were excluded. We did not expect involvement based entirely on the patients’ or relatives’ initiative to be successful. Instead the patients were convenience sampled based on a clinical eligibility assessment made by three experienced medical doctors who invited the eligible patients to be interviewed. We did not involve patients who were considered at risk of exhaustion, further functional decline or of delirium or any other harmful event by being involved. In case of the development of delirium, the patient was excluded from further involvement. Frailty status was assessed using the MPI, which defines frailty as a MPI score > 0.33. Recognising the value of the relatives’ experiences and to support the patients, patients’ relatives were also encouraged to join if the patient consented. The patients and their relatives were provided with a booklet (Additional file 1) that described the intended involvement process and PROM in lay terms. The information material was written by collaboration between the HCPs. The HCPs (two medical doctors (EMD, MEJJ) and one specialist nurse (MG) engaged in frailty- and transitional care research) were reinvolved at the end of data collection. The number of HCPs was deliberately lower than the number of patients and relatives to address potential power differentials.

### Involvement level and involvement steps

Several involvement evaluation models have been developed [18, 38]; however, there is no consensus on a uniform model that can objectively describe the degree of involvement in research. We found the Health Canada Public Involvement Continuum (HCPIC) [16, 20] useful and easy to understand; the model describes patient involvement as a continuum divided into five levels, where all involvement levels are valuable and the different levels meet the different needs and capabilities among those involved. The model illustrates the increasing level of collaboration between researchers and the involved people as the involvement moves from unidirectional communication and consulting towards involvement in a co-productive partnership.

**Fig. 1:**
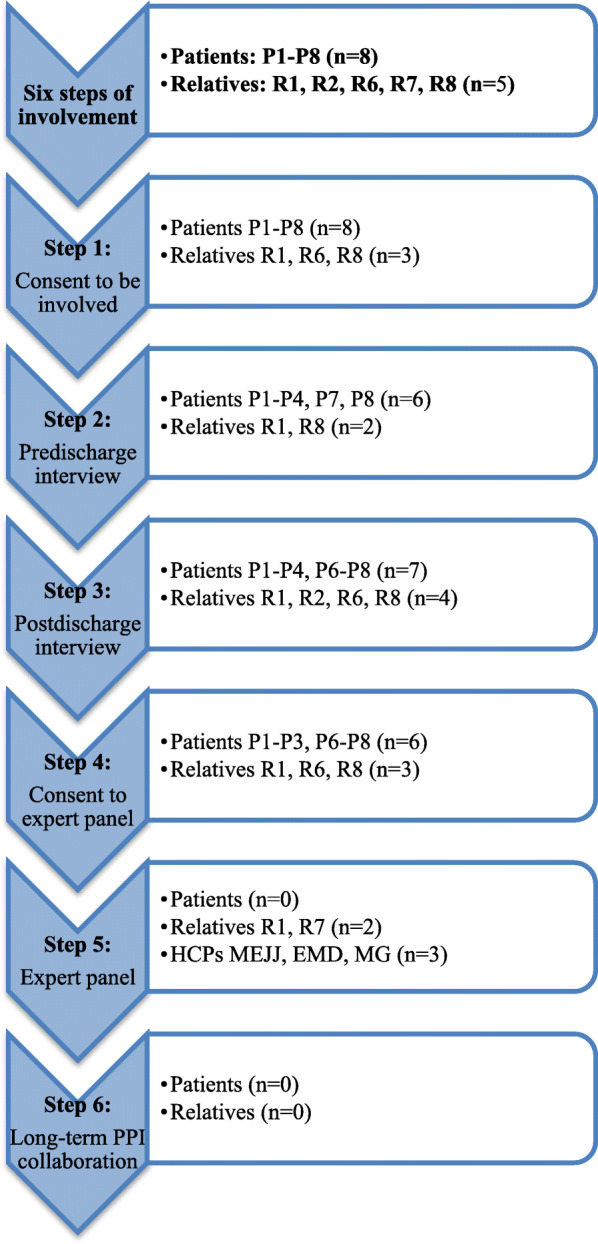
Flow of involved patients, relatives and HCPs. The eight patients are named P1–P8. The involved relatives are named Rx, x being the number of the corresponding patient to whom they are related. R2 was involved starting from the second interview, and R7 was involved starting at the expert panel meeting. R7 offered to stand in for P7. Two patients dropped out before the first interview; however, P6 was involved again in the second interview, and R6 was involved there as well. One patient (P4) and one relative (R2) dropped out after the second interview. The remaining six patients and three relatives all gave consent to be involved as members of the expert panel; however, all except R1 dropped out before the panel meeting. Abbreviations: HCPs: health care professionals; PPI: patient and public involvement

Given the involved patients’ frailty, a pragmatic involvement approach was needed. To achieve the maximum level of involvement and adherence during the study, the study frame allowed for continuous recruitment and drop-in and drop-out throughout the research process [39]. Six steps were defined based on the intended course of the study (Fig. 1). The first step was to ensure patient involvement and consent to further involvement. Prior to each step, oral consent was reverified. The second step was to conduct individual interviews while the patients were still in the hospital. The third step was to conduct postdischarge interviews in the patients’ homes, nursing homes or rehabilitation centres. The fourth step was to organise an expert panel meeting with the attendance of patients and relatives. The fifth step was to conduct the expert panel meeting and involve the panel members in the data analysis and development of PROMs. The final step was to seek to enter into a long-term collaboration and involve patients and relatives in new research projects as research partners.

### Collecting data: interviews and reflective notes

The purpose of the interviews was to clarify the patients’ experiences during discharge, thus providing data for the subsequent thematic analysis [40]. Data consisted of pre- and postdischarge interviews with patients and relatives, as well as the interviewer’s reflective notes.

The patients were interviewed in their single-bed room on the day before discharge. A relative was also interviewed if the patient desired. The interviewer (TKH), who is a PhD student and specialty registrar in geriatric medicine, presented himself as the study’s contact person before informally introducing the study, the objectives of being involved and the interview subject. The interviewer explicitly underlined that the talk should not concern the patient’s medical history. The aim was not to evaluate the previous care or caregivers but rather to have a casual talk about subjects of relevance to the patient regarding the concept of being an inpatient awaiting discharge. The interview concept allowed flexibility for dialogue. None of the interviews were limited by time, allowing for a relationship to form between the interviewer and interviewees. To maintain confidentiality and a trustful atmosphere, the interviews were not audio recorded. The patients and relatives could stop the interview at any point. The predefined probing questions asked were as follows: *‘In your opinion, what is most important to ensure a successful discharge from the hospital?’* and *‘What could be done to make you feel secure during discharge and the following days?’* The researcher noted the keywords and sentences mentioned by the patients and relatives during the interview, as well as the researcher’s own reflections that emerged as the interview proceeded.

The patients and relatives were then offered a second interview after discharge, which was either performed in the patient’s residence or on the phone. An appointment was scheduled to take place between two to four days after discharge. The interviewer resumed the keywords and topics from the first interview. The focus of the second interview was the discharge process and transitional care as a whole given the new circumstances of having left the hospital environment. Three predefined questions were asked, as follows: *‘How do you feel, now that you are at home (if the patient was discharged home)?’, ‘Do you wish anything to be different?’* and *‘Has anything changed regarding your view on the discharge process since our last talk?’* The interviewer again made notes as appropriate during the talk. By the end of the second interview, the patients and interviewed relatives were invited to participate in an expert panel meeting back at the hospital later on. If accepted, a reminder phone call was scheduled.

### Data analysis: theme formation and expert panel

Data were manually analysed by the interviewer using a thematic analysis [41]. A pseudomised written summary was made based on the interview keywords. Emerging categories were then grouped in themes, and each were elaborated on using descriptive text. Subsequently, all interviewees were invited to form an expert panel together with the HCPs, further developing the derived themes and drawing up PROMs for future use. The expert panel meeting took place on April 9, 2019. Along with the invitation, the themes were listed in lay terms. The patients and relatives were offered transportation, further information and other supportive arrangements, if needed. They were also allowed to select a stand-in representative or to bring a companion. The meeting took place in the hospital in a calm conference room away from the ward the patients had previously been admitted to. The interviewer acted as a facilitator, welcoming the panel members and emphasising the importance of an open and equal dialogue. The attendees were offered refreshments. The meeting was scheduled to last approximately two hours, but this time limit was flexible. The panel was given two main tasks: 1) to discuss, rank and validate the identified themes and 2), if possible, to establish PROMs based on the findings. The discussion was based on a short introduction of each theme. The themes were printed in individual colours and scattered on the table like pieces in a puzzle, encouraging those present to single out the most important themes. The panel was also asked to validate each theme. Based on the dialogue, the panel summarised the discussion and proposed PROMs. Before wrapping up the meeting the non-HCP panel members were invited to be involved in further research as part of a steering committee in future research projects. After the meeting, all the attending panel members were invited to comment on the written summaries and the PROMs, which were elaborated on by one of the HCP panel members (MG) and the interviewer.

To assess the degree of equality and the benefits of cooperation between the patients, relatives and researchers in the involvement process, the non-HCP participants later received a prepaid envelope and a printed questionnaire to be filled in at home (Additional file 2).

### Ethical considerations

All patient data regarding MPI frailty level was derived from an ongoing quality development project approved by the Regional Research Ethics Committee, Central Denmark Region (journal no. 197/2017). No patient consent form was needed, and referral was not required. All participants could decline being involved at any point.

## Results

### Involved people and involvement steps

The group of interviewees was composed of eight patients and five relatives. The average patient age was 86 years (range 75 to 94 years), five were female, and their mean MPI score (range: 0–1; score 0–0.33: non-frail; 0.33–0.66: moderately frail and 0.66–1.0: severely frail) was 0.65 (range: 0.44–0.75). No data were routinely collected regarding the relatives or the three HCPs; however, the relatives were two sons and three daughters, all middle aged. One had previous experience as a patients committee member. The flow of involved people along with the corresponding steps is displayed in Fig. 1.

All of the invited patients agreed to be involved in the study. Eight patients and three relatives agreed to receive a postdischarge visit and interview (step 1). One patient (P5) became delirious before the first interview, while another (P6) could not take part without a relative (R6) because of a language barrier. Others felt unsure without their relative. Accordingly, for at least three of the patients, patient participation at any step would not have been accomplished if the relatives had not been present. A total of eight in-hospital (step 2) and 10 postdischarge interviews (step 3) were performed. Five patients were interviewed at home and one in a rehabilitation centre (P4). Three of the relatives were present at the second interview (R1, R6, R8) on request from both the patient and the relatives. Besides being frail, P2 was afflicted with grief. P2 did not want to burden his relatives; hence, the existence of R2 had remained unspoken of throughout the admission phase. R2 was interviewed on the phone after P2’s second interview. After the second interview, all interviewees except one (P4) willingly agreed to be invited to be a part of the expert panel meeting (step 4) to be held approximately one month later. However, P2 did not allow further involvement of R2. Furthermore, all apart from one (R1) individual cancelled shortly before the meeting. Five patients (P1, P2, P3, P6, P7) felt unfit to leave home while one (P8) was now receiving palliative care, so R8 sent his apologies. Another relative (R6) happened to be unable to go on the specific date. Having previously been involved in another research project, yet not previously involved in the present study, the relative of one of the patients (P7) offered to step in despite the late stage of the study. The expert panel (step 5) now consisted of two relatives (R1, R7; one son, one daughter); one senior doctor/professor in geriatric medicine (EMD); one clinical specialist nurse/researcher (MG); and one junior doctor specialist in geriatric medicine (MEJJ). At the meeting, both relatives agreed to continue involvement as members of a future transitional care research steering committee, which would consist of two annual meetings for the next three years. However, shortly after the expert panel meeting, one of the patients passed away, and the relative no longer found it meaningful to be involved. Neither of the patient-representing expert panel members responded to the evaluation questionnaire after the expert panel meeting, and the intended long-term involvement (step 6) petered out during the following months.

### Level of involvement

Achievement of the five HCPIC levels of involvement varied as the study proceeded. All of the invited patients and relatives readily agreed to be involved (HCPIC level 1). The interviews were characterised by data collection and conversation and interaction between the interviewer and interviewees, thus raising the level of involvement to delivering information and acting as consultants (HCPIC levels 2–3). The expert meeting suffered from patient drop-out (Fig. 1); nevertheless, the involvement level of the remaining relatives was very high. Indeed, they and the HCPs were highly engaged (HCPIC level 4) in discussing and analysing the data and forming PROMs. The final, intended long-term, partnership involvement level (HCPIC level 5) was not reached because none of the patients and relatives wished to remain involved.

### Themes and PROMs

The expert panel agreed that the data and resulting themes were thorough and valid in expressing the patients’ positions and views. The 16 themes were divided and prioritised based on six headlines, as displayed in Table 1.

**Table 1:** The prioritised themes expressed by frail older patients and relatives going through a care transition from the hospital to their own home or rehabilitation centre

Headlines (prioritised by importance)	Themes discussed by the expert panel					Not actively prioritised by the expert panel
**Care contents: overview and responsibility**	Health care personnel competency, options and drive: Granted services vs. the ability to deal with current individual needs	Identify and solve practical challenges	Number of carers/ health care professionals: too many/few/late/early/often or wrong profession			
**Relatives: involvement in care decisions**	Involvement of the relatives during admission: consulting the relatives’ views	Involvement in care planning: participating relatives, assigning tasks to the relatives	Relatives taking an active part in the transition: being present at discharge	Relieve burdens off relatives and avoidance of overloading	Practically possible to be involved (time, place etc.)	
**Care transition: overview, responsibility**	Manager/coordinator: to clarify and define responsibilities	Knowledge about options, e.g., whom to contact in case of unforeseen events				Match care and treatment plan expectations to reach common agreement
**Existential issues**	Existential and emotional considerations and reflections during admission, transition and after discharge					
**Functional capacity, illness and disease**	Physical functional capacity and social capacity: regaining loss of function	To return home				Diagnostic conclusion: to understand what happened
**Culture**						Cultural understanding Language barrier

Most importantly, the top priorities were related to care and practicalities, the transparency of the transition of care process, division of responsibilities and the role of relatives. The expert panel confirmed that individualised involvement of relatives during admission and discharge is desired and highly appreciated among patients, relatives and HCPs. A substantial amount of time was spent discussing existential issues, that is, resuscitation attempts in the case of cardiac arrest, advanced care planning and end-of-life decision making. The panel attendees did not reach an agreement on whether or not resuscitation should routinely be discussed during hospitalisation; however, they did agree that the topic itself was very important, especially regarding frail older people. The themes concerning functional recovery, returning home and patients’ understanding of the course of disease were not highly prioritised, and the cultural issues were not actively prioritised at all.

The discussion of the 16 themes resulted in seven PROM proposals (Table 2). Four of the proposals (1–4, Table 2) were designed as questionnaires intended to support clinical practice directly as dialogue-triggering instruments applied to improve the quality of transitional care. Moreover, readmission, recurrence of illness and proper discharge letters pointing out further available treatment options were considered as relevant quality of care measures for patients, relatives and HCPs.

**Table 2:** PROM measures established during the expert panel meeting

	PROM	Who?	When?
1.	Postdischarge questionnaire for relatives to fill in: 1. ‘Are you confident, that drugs are administered safely?’ 2. ‘In your opinion, are the nutritional and fluid needs taken care of safely/properly?’ 3. ‘Are you satisfied with the arrangements made?’ 4. ‘Are you satisfied with the quality of the arrangements’ 5. ‘Are the arrangements tailored to the patient’s needs? (Regarding circadian rhythm, individual hygiene requests, and flexibility in services)?’ 6. ‘Are your tasks as relatives proper and fair?’	Relatives. If no relatives are present, the nearest care provider or a friend may act as a proxy	After discharge
2.	Existential reflections questionnaire: 1. Has the life situation been discussed at any point? 2. Do the patient and/or relatives wish to discuss the situation further? 3. Does the patient want to be admitted to hospital in the future? (Yes/no/depending on)	Patients and/or relatives together with Health care professionals (doctors)	According to the patients and/or relatives wishes. Appropriate timing and setting is essential. Even so, the subject should be addressed during admission or discharge
3.	Home facilities and assistive remedies questionnaire: 1. ‘Were the assistive remedies ready and in place at discharge?’ 2. ‘Is the patient able to use the remedies?’ 3. ‘Is the current home the right place?’	Relevant health care professionals Relatives Patients	Shortly after care transition
4.	The importance of having involved relatives: 1. ‘Were your relatives involved sufficiently and appropriately during the care transition?’ 2. ‘Were you sufficiently and appropriately involved during your parent’s/ cohabitant partners’ care transition?’	Patients Relatives	During care transition
5.	Other relevant outcome measures: • Readmission/recurrence of illness	Researchers	Postdischarge
6.	• Caregiver burden questionnaire • Quality of life questionnaire • Loneliness questionnaire	Health care professionals together with patients and/or relatives	After discharge
7.	• Available treatment options should be pointed out in the discharge letter	Doctors	At discharge

Abbreviations: *PROM* patient-reported outcome measures

## Discussion

The aim of involving frail older patients in research was achieved to a moderate (HCPIC level 3) extent. To raise the involvement level above the consulting level, we had to rely on relatives and HCPs. However, using HCPIC we did not reach beyond level four with the public. The frailty of the patients meant that it meant a lot of work for relatives to take care of the patients. The involvement process produced PROMs which were well founded, bringing into focus the views of the frail older patients.

Though the involved patients and relatives willingly took part in the involvement and interviews, we faced several challenges during the process. First, we deliberately chose to involve the fittest frail patients who had recently experienced a care transition. By coincidence, none of the involved patients were nursing home residents, and only one patient was discharged to a rehabilitation centre. Nonetheless, as reflected in the MPI score, the patients all had high frailty scores for multiple reasons. None of them were completely self-sufficient after hospital discharge. We consider the involved patients as typical for the population in focus: all of them being older, frail and exposed to challenging care transitions. Second, involvement had to be tailored to the patient’s needs; despite this, some patients were excluded, and many dropped out at an early stage. Drop-outs probably introduced some distortion, especially in prioritising the themes and elaborating on the PROMs because none of the patients took part in the expert panel. Also there was a lack of representation of persons with Danish as a second language amongst the expert panel. Nonetheless, two highly capable relatives were present and were discussing what the patients had earlier expressed. The need to transport frail older patients back to the hospital shortly after discharge resulted in none of these patients accepting to attend the expert panel meeting. Third, not all of the patients fully understood or remembered the reasons for involving them in the research; nonetheless, all of them contributed to the results by sharing their points of view, as supported by their relatives. Postponing involvement further, the patients may not recall clearly what happened.

Contrasting our involvement approach, other studies relating to care transitions in older people involved community-dwelling, younger participants in postacute settings [27, 42, 43]. Heaven et al. [44] recommend recruiting core group public members from established voluntary community groups to provide continuity over time and to facilitate recruiting other public members along the way. Involving such volunteers may ensure high involvement levels and continuity; however, it may also reduce the level of lived experience among the involved people. In our study, the relatives, HCPs and patients did not always agree, stressing the importance of involving the frail patients (and relatives) themselves. Recruiting patients, relatives and other stakeholders seems essential to achieve both long-term participation and first-hand experiences.

The HCPIC was used to measure the involvement level in our study, absorbing and linking participation and PPI in a continuum. Viewing involvement as a continuum allowed us to involve frail older patients and their relatives during stressful times. However, it also posed a risk of pseudo-involvement and of doing research “about” patients rather than “with” or “by” them, stressing the importance of separating “PPI” and “research involvement” from “study participation”. We believe the HCPIC is a useful approach to research involvement with vulnerable patients. However, relatives as well as other stakeholders should also be involved to achieve higher involvement levels, e.g. formation of long-term collaborative research groups. Traditional PPI could have provided additional benefits.

Many of the desired components of transitional care revealed by the patients and relatives in the present study have previously been described by others [25–27, 42]. Dyrstad et al. [3] report on the relatives’ important role as advocates for their family members during hospital admission and discharge. Similarly, we found relatives playing an invaluable role in supporting the patients during the interviews, helping them remember what happened. Likewise, several of the PROM results concerned the role of the relatives during the care transition. Notably, the PROM questionnaires proposed by the expert panel (Table 2, 1-4) were intended to serve both as transitional care quality measures, study outcome measures (i.e., PROMs) and as precautionary measures to ensure good practice of involving the relatives in care transitions. Nyborg et al. [45] argue that user participation in care transitions is a family matter; correspondingly, our results suggest that involvement of frail older patients in research is a family matter, too. Involving relatives might entail some limitations as well: the relatives of frail patients were readily involved and played a particularly important role in enhancing research involvement, as well as successful care transitions of the frail patients in focus. Nevertheless, not all patients wanted their relatives to be involved; some patients had no relatives, or their relatives were unable to participate; and some ceased to partake in further involvement once their loved ones were no longer involved.

None of the involved commented on the themes before the expert panel meeting, nor did the relatives return the evaluation questionnaire regarding their perceptions of being involved. We did not beforehand make any formal agreement with the involved regarding these tasks, and the intended follow-up tasks (comment on themes, fill out questionnaire) may have exceeded the capacity of the involved patients and relatives.

The thematic analysis was made without using computer-assisted qualitative data analysis software, and to avoid interrupting the trustful atmosphere, the data were not audio recorded and transcribed [40]. This represents a limitation of the current study; still, the dataset was manageable and the resulting themes were post-hoc validated by relatives and the HCPs who had been working with older people for several years. Given the reasonable information power the interview data contained, we consider the number of interviewed patients sufficient [37].

## Conclusion

In conclusion, our study showed that involving frail older patients in research during care transitions was feasible when choosing a pragmatic approach; however, involvement in terms of forming a PPI panel was not achieved. Involving patients with substantial physical or cognitive impairment during care transitions is challenging, and involving their relatives is paramount. Despite the limitations, we argue that the involvement of patients, relatives and other stakeholders holds the potential to become an inherent and valuable part in geriatric, frailty and transitional care research studies. A set of transitional care PROMs for frail older patients was proposed for further validation.

Our advice for the future research involvement of frail older patients is as follows:

1. Carefully consider the balance between the value of lived experiences and the involved person’s ability to actively be involved for a longer period of time. It may be worthwhile involving people at risk of a condition in the future or people with past experiences in addition to, or if necessary instead of, those currently experiencing the condition.
2. The value of involving relatives cannot be overstated although the risk of biased opinions must also be considered and addressed.
3. Make involvement easy and allow the involved to de- and re-engage. Consider continuous or repeated involvement of new people, and remain open to involvement at different levels.
4. Avoid pseudo-involvement: you want to move beyond the level of participation.
5. Reach out to the patients and relatives where they are.
6. Discuss and agree upon the extent and duration of involvement from the very beginning. Also, agree on how to stay in touch.
7. Involve people during study planning, first by defining the future involvement approach: how, why, when and who to involve during the research cycle should be decided in collaboration with patients, relatives and/or other laypersons.

## Supplementary Information

**Additional file 1.**

**Additional file 2.**

## Data Availability

The dataset generated and analysed during the current study is not publicly available because of obligations to maintain confidentiality, but anonymised data are available from the corresponding author on reasonable request.
