# Supplementary material for: Involving frail older patients in identifying outcome measures for transitional care—a feasibility study
Source: Res Involv Engagem. 2021 Jun 3;7:36. doi: 10.1186/s40900-021-00288-9 (PMC8173811; doi:10.1186/s40900-021-00288-9)
Supplement: Supplementary file 1 — Additional file 1. [file 40900_2021_288_MOESM1_ESM.docx]

##### **‘Meaningful outcomes during hospital discharge’**

# ‘Meaningful outcomes during hospital discharge’

***Dear attendee!***

***Thank you for your willingness to be involved in two informal interviews regarding my research project***

I would like to talk to you about your experiences and perceptions regarding your upcoming hospital discharge. The aim is to gain insights into how you and your relatives feel during a care transition.

The conversations are NOT supposed to be about your current health issues; instead, the focus should be on the situation you are currently in: awaiting hospital discharge.

I am used to seeing things from a doctor’s perspective. As a part of my research, I am keen to know what matters to you

My PhD project deals with home visits offered to frail older people during and after hospital discharge. We are investigating the impact of home visits on various outcomes. These outcomes are chosen by the researchers. One example using blood pressure as a study outcome is displayed below:

Example: If a researcher is interested in evaluating the effect of an antihypertensive drug, he/she will measure the blood pressure. However, patients suffering from hypertension rarely sense any symptoms. What really matters to the patient may be completely different things such as pill size, number, prize etc.

***I am interested in your opinion on what outcomes should be used when studying care transitions***

I will be asking you a few questions while jotting down notes during our conversation. I am interested in what is needed for you to feel safe during the forthcoming care transition. Later, we will analyse the answers and propose outcomes to be used in future research.

In a few days, I would also like to call you on the phone or visit you at home (depending on your preference) to ask a few more questions.

***Opportunity to be involved as a working group member***

In total, nine people will be interviewed in the same way as you. You are all invited to be involved in a working group. The working group will be invited to a meeting where the results based on the interviews will be presented. When the PhD study is completed in about two and a half years, you will all be invited to another meeting.

The working group’s purpose is to be ‘curious, inquisitive and inquiring allies’ commenting on the research project in a confidential atmosphere. If requested, we will offer free transportation related to the meetings, and refreshments will be provided. The meetings are scheduled to last approximately one hour.

It is completely up to you whether you want to be involved in one or two interviews and/or one or two meetings.

###### Involvement is voluntary

You can stop your involvement any time during the interviews and meetings. Involvement is absolutely voluntary. No matter how much you wish to be involved, the interviews will not influence treatment or care during hospitalisation, care transition or postdischarge care. All interviews are anonymised, and the talks are not audio recorded. Being a doctor and researcher, I am subject to confidentiality similar to the other health care professionals you have met during the admission.

If you wish to know more about the PhD project, a scientific protocol is publicly available (in English) on [www.clinicaltrials.gov](http://www.clinicaltrials.gov) (ID-number NCT03796923).

#### If you have any questions regarding the project, you are welcome to contact me at any time!

#### Kind regards

Troels Kjærskov Hansen

MD, PhD-student

Department of Geriatrics, Aarhus University Hospital

Aarhus University

e-mail: [trohas@rm.dk](mailto:trohas@rm.dk)
