# Supplementary material for: Involving frail older patients in identifying outcome measures for transitional care—a feasibility study
Source: Res Involv Engagem. 2021 Jun 3;7:36. doi: 10.1186/s40900-021-00288-9 (PMC8173811; doi:10.1186/s40900-021-00288-9)
Supplement: Supplementary file 2 — Additional file 2. [file 40900_2021_288_MOESM2_ESM.docx]

Dear X

During the working group meeting at Aarhus University Hospital in April, we agreed to send out a questionnaire as part of the research involvement project ‘Meaningful outcomes during hospital discharge’.

Hence, I would like you to fill out the scheme below and return it to me in the prepaid envelope. If necessary, please elaborate on the back of the paper.

We are expecting our collaborative work will to be turned into a research paper (in English); hopefully, it will be ready for you to read in about 8–12 months.

On behalf of the other researchers,

Kind regards

Troels K Hansen,

MD, PhD-student

Department of Geriatrics

Aarhus University Hospital

**1 Why did you chose to be involved?**

**2 How was it to be involved?**

**3 Did you think of the working group members as equal?**

**4 Did your involvement prompt you or your family members considerations?**

**5 How did you profit from being involved?**

**6 Do you have other thoughts you wish to share regarding your involvement?**

**7 Do you consider involvement in research meaningful?**
